# Serum retinol-binding protein 4 in stroke patients: correlation with T helper 17/regulatory T cell imbalance and 3-year cognitive function decline

**DOI:** 10.3389/fneur.2023.1217979

**Published:** 2023-09-21

**Authors:** Fei Wang, Yaqin Qin, Zongyou Li

**Affiliations:** Department of Neurology, Fuyang People’s Hospital, Fuyang, China

**Keywords:** stroke, retinol-binding protein 4, T helper 17/regulatory T imbalance, cognitive impairment, cognitive function decline

## Abstract

**Objective:**

Retinol-binding protein 4 (RBP4) promotes atherosclerotic progression and neuronal loss, whereas its association with cognitive impairment in stroke is unclear. Hence, this prospective study aimed to explore the association of serum RBP4 with the T helper (Th)17/regulatory T (Treg) cell ratio and its correlation with cognitive impairment in stroke patients.

**Methods:**

Peripheral blood samples from 265 stroke patients and 50 healthy controls (HCs) were collected at enrollment for serum RBP4 (by enzyme-linked immunosorbent assay) and Th17 and Treg cells (by flow cytometry) determination. Additionally, stroke patients underwent routine follow-ups, and their Mini-Mental State Examination (MMSE) scores were assessed at baseline and in years 1, 2, and 3 after enrollment.

**Results:**

Serum RBP4 was elevated in stroke patients compared to HCs (*p* < 0.001), with a good ability to differentiate stroke patients from HCs (area under the curve: 0.815). Serum RBP4 was positively associated with Th17 cells (*p* < 0.001) and the Th17/Treg cell ratio (*p* < 0.001) and negatively associated with Treg cells (*p* = 0.003) in stroke patients, whereas it was only positively associated with the Th17/Treg cell ratio (*p* = 0.027) but not with Th17 (*p* = 0.075) or Treg (*p* = 0.130) cells in HCs. Furthermore, increased serum RBP4 was associated with a lower MMSE score (*p* < 0.001) and a lower incidence of cognition impairment (*p* = 0.005) at enrollment in stroke patients, as were Th17 cells and the Th17/Treg cell ratio (all *p* < 0.050). The 1-, 2-, and 3-year MMSE scores in stroke patients were 25.9 ± 2.0, 25.3 ± 2.3, and 24.9 ± 2.3, respectively. More importantly, serum RBP4 was negatively correlated with 1-, 2-, and 3-year MMSE scores (all *p* < 0.001) and positively associated with 1-year (*p* = 0.013), 2-year (*p* = 0.007), and 3-year (*p* = 0.001) MMSE score declines in stroke patients.

**Conclusion:**

Serum RBP4 is positively associated with a Th17/Treg cell imbalance and, more importantly, it is indicative of cognitive function decline within 3 years in stroke patients. Thus, early and timely interventions and physical rehabilitation are more necessary in stroke patients with high serum RBP4.

## Introduction

Stroke, including ischemic and hemorrhagic subtypes, is a common cerebrovascular disease characterized by high disability and fatality (1, 2). It is estimated that there are 2.4 million new cases of stroke and 1.1 million stroke-related deaths in China each year (3). At present, the standardized comprehensive treatment for ischemic stroke mainly involves intravenous thrombolysis, endovascular thrombectomy, antiplatelet therapy, etc. (4, 5). For the treatment of hemorrhagic stroke, craniopuncture, blood pressure and elevated intracranial pressure management, etc., are generally applied depending on the specific situation (6). Despite the progress and advancement in stroke treatment, the outcome of stroke is still not ideal, with cognitive impairment in over 70% of stroke survivors (7–9). At present, the management of post-stroke cognitive impairment mainly includes the following two aspects: pharmacological treatment (for example, with cholinesterase inhibitors, memantine, cerebrolysin, etc.) and cognitive rehabilitation; nonetheless, the occurrence and exacerbation of cognitive impairment are still prevalent and increase the risk of disability (8, 10). Consequently, seeking biomarkers to identify stroke patients at high risk for cognitive dysfunction is helpful to improve their rehabilitation and prognosis.

Retinol binding protein 4 (RBP4), a transport protein belonging to the lipocalin family, is implicated in the incidence and progression of atherosclerosis by inducing macrophage-divided foam cell formation and regulating the Janus kinase 2/signal transducer and activator of transcription 3 (JAK2/STAT3) signaling pathway (11–14). From a clinical point of view, a previous study suggested that higher RBP4 was associated with increased disease severity and a worse physical and functional outcome in acute ischemic stroke (AIS) patients (15). Another study found that AIS patients with lower RBP4 were more likely to experience disease progression and disability (16). However, the correlation of RBP4 with cognitive impairment induced by multiple factors (such as the imbalance of T helper (Th)17 and regulatory T (Treg), neuronal loss, etc.) has rarely been reported in stroke patients thus far. Notably, in addition to facilitating atherosclerosis development, RBP4 also facilitates neuronal loss (17–19). For instance, one study revealed that RBP4 regulates cortical and hippocampal (CA3) neuronal loss (18). Another study showed that RBP4 aggravates neuronal loss by modulating lipoprotein-associated phospholipase A2 (Lp-PLA2) and netrin-1 (17). Conceivably, RBP4 is speculated to possess a predictive role for cognitive impairment in stroke patients.

Therefore, this prospective study aimed to investigate the association of serum RBP4 with Th17/Treg balance, disease characteristics, and the long-term progression of cognitive impairment in stroke patients.

## Materials and methods

### Participants

A total of 265 stroke patients were consecutively enrolled between March 2019 and January 2020. Inclusion criteria were as follows: (i) diagnosed with stroke according to an American Heart Association/American Stroke Association (AHA/ASA) guideline (20) and (ii) age > 18 years. Exclusion criteria were as follows: (i) malignant disease, (ii) immune system disease or active infection, (iii) global or receptive aphasia, (iv) intracranial hemorrhage, and (v) lactation or pregnancy. In addition, a total of 50 healthy subjects with no abnormalities on recent physical examination were included as healthy controls (HCs) if they met the following conditions: (i) age- and gender-matched with stroke patients; (ii) without a malignant disease; (iii) without a history of stroke or subclinical stroke; (iv) without an immune system disease or active infection; and (v) not pregnant or lactating. This study was approved by the Ethics Committee of Fuyang People’s Hospital, and informed consent was obtained from each participant or guardian.

### Data and sample collection

Characteristics that included demographics, comorbidities, and disease-related information were collected from stroke patients. Peripheral blood (PB) samples were collected from all participants (stroke patients, collected at admission or revisit; HCs, collected after enrollment). Each PB sample was divided into two parts: one part was used to extract serum for RBP4 detection, and the other part was used for Th17 and Treg cell detection.

### RBP4 detection

Serum RBP4 was determined by the enzyme-linked immunosorbent assay (ELISA) method using Human RBP4 Quantikine ELISA Kits (No. Cat. DRB400, R&D Systems, Inc., Minneapolis, Minnesota, United States). The experimental steps are briefly summarized as follows: first, standards or samples were added to the precoated microplates and incubated for 1 h at 37°C. Next, the antibody conjugate was added and incubated for 1 h at 37°C. After washing, substrate solution was added and further incubated for 30 min at 37°C. Finally, stop solution was added, and the intensity of the color was read at 450 nm within 30 min using a Multiskan spectrometer (Thermo Fisher Scientific, Inc., Waltham, Massachusetts, United States). Samples were tested in triplicate.

### Th17 and Treg cell detection

First, CD4^+^ T cells were separated from the PB samples using a Dynabeads™ FlowComp™ Human CD4 kit (No. Cat. 11361D, Thermo Fisher Scientific, Inc., Waltham, Massachusetts, United States). Then, the proportions of Th17 and Treg cells in CD4^+^ T cells were determined by flow cytometry (FCM) using the FlowX Human Th17 Cell Multi-Color Flow Cytometry Kit (No. Cat. FMC007B, R&D Systems, Inc., Minneapolis, Minnesota, United States) and the Regulatory T Cell (Treg) Flow Cytometry Panel (No. Cat. FMC-P-004, R&D Systems, Inc., Minneapolis, Minnesota, USA). The Th17/Treg ratio was then calculated. All tests were performed in strict accordance with the reference method of the kit used. Samples were tested in triplicate.

### Follow-up and evaluation

Stroke patients underwent routine follow-ups. Patients’ cognitive function was assessed using the Mini-Mental State Examination (MMSE) score at enrollment and every year thereafter. The highest MMSE score was 30, and a score of less than 27 was considered to indicate cognitive impairment (21). The 1-, 2-, and 3-year declines in MMSE scores were defined as the MMSE score at the 1^st^/2^nd^/3^rd^ year after enrollment minus the MMSE score at enrollment, respectively. During the follow-up period, 42 (15.8%) patients were lost to follow-up, and 17 (6.4%) patients died. Missing values from these patients were treated as vacancies.

### Statistics

SPSS v.22.0 (IBM, Inc., Amenk, New York, United States) and GraphPad Prism v.7.0 (GraphPad Software, Inc., San Diego, California, United States) were used for data processing and figure generation, respectively. The Mann–Whitney U test and Kruskal–Wallis H test were used for comparative analysis. A receiver operating characteristic (ROC) curve demonstrated the ability of serum RBP4 to distinguish between stroke patients and HCs. A backward stepwise multivariable logistic regression model was constructed to explore the prognostic ability of serum RBP4, and the ROC curve was then used for validation. Spearman’s test was utilized for correlation analysis. *p* < 0.05 was considered significant.

## Results

### Characteristics of stroke patients

Stroke patients comprised 103 (38.9%) women and 162 (61.1%) men, whose mean age was 68.9 ± 8.2 years. Regarding comorbidities, 214 (80.8%), 131 (49.4%), 79 (29.8%), 61 (23.0%), and 110 (41.5%) patients had hypertension, hyperlipidemia, diabetes, chronic kidney disease (CKD), and cardiovascular disease (CVD), respectively. Additionally, 180 (67.9%) patients were diagnosed with a first stroke, and the remaining 85 (32.1%) patients were identified as having a recurrent stroke. The mean MMSE score at enrollment was 26.5 ± 1.9. A total of 105 (39.6%) patients were assessed as having cognitive impairment at enrollment. Specific information on stroke patients is shown in Table 1.

**Table 1:** Characteristics of stroke patients.

Characteristics	Stroke patients (*N* = 265)
Age (years), mean ± SD	68.9 ± 8.2
Gender, No. (%)	
Women	103 (38.9)
Men	162 (61.1)
Educational level, No. (%)	
Primary school or less	106 (40.0)
Middle or high school	118 (44.5)
Undergraduate or above	41 (15.5)
Marital status, No. (%)	
Married	133 (50.2)
Single/divorced/widowed	132 (49.8)
Place of residence, No. (%)	
Urban	226 (85.3)
Rural	39 (14.7)
History of smoking, No. (%)	
No	152 (57.4)
Yes	113 (42.6)
Hypertension, No. (%)	
No	51 (19.2)
Yes	214 (80.8)
Hyperlipidemia, No. (%)	
No	134 (50.6)
Yes	131 (49.4)
Diabetes, No. (%)	
No	186 (70.2)
Yes	79 (29.8)
CKD, No. (%)	
No	204 (77.0)
Yes	61 (23.0)
CVD, No. (%)	
No	155 (58.5)
Yes	110 (41.5)
Lesion location, No. (%)	
Left	95 (35.9)
Right	95 (35.9)
Bilateral/brainstem/unknown	75 (28.2)
Diagnoses, No. (%)	
First stroke	180 (67.9)
Recurrent stroke	85 (32.1)
MMSE score at enrollment, mean ± SD	26.5 ± 1.9
Cognitive impairment at enrollment, No. (%)	
No	160 (60.4)
Yes	105 (39.6)

SD, standard deviation; CKD, chronic kidney disease; CVD, cardiovascular disease; MMSE, Mini-Mental State Examination.

### Comparison of serum RBP4, Th17, and Treg cells between stroke patients and HCs

**Figure 1:**
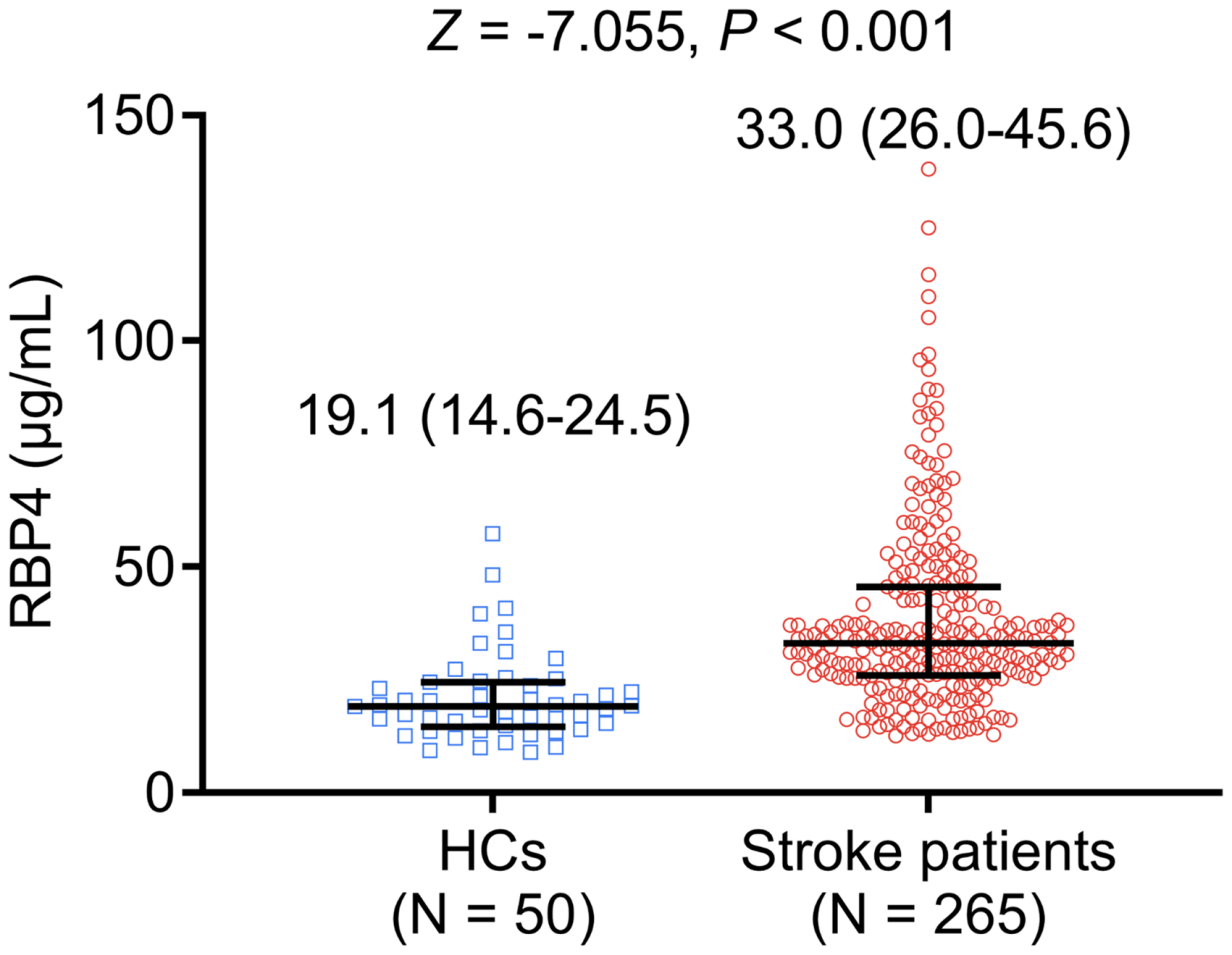
Comparison of serum RBP4 between stroke patients and HCs.

Serum RBP4 was elevated in stroke patients compared to HCs [median interquartile range (IQR): 33.0 (26.0–45.6) μg/mL vs. 19.1 (14.6–24.5) μg/mL, *p* < 0.001] (Figure 1). Meanwhile, serum RBP4 had a satisfactory value to distinguish stroke patients from HCs, with an area under the curve (AUC) value of 0.815 (95% confidence interval: 0.750–0.879). The best cut-off value of serum RBP4 was 25.5 μg/mL (sensitivity: 0.762, specificity: 0.820) (Supplementary Figure S1).

Th17 cells were increased (*p* < 0.001), but Treg cells were decreased (*p* < 0.001) in stroke patients compared to HCs. Subsequently, the Th17/Treg ratio was elevated in stroke patients compared with HCs (*p* < 0.001) (Table 2).

**Table 2:** Comparison of Th17 cells, Treg cells, and Th17/Treg ratios between stroke patients and HCs.

Items	HCs (N = 50)	Stroke patients (N = 265)	*Z*	*p* value
Th17 cells (%), median (IQR)	1.8 (1.1–3.1)	3.9 (3.0–5.6)	−7.801	<0.001
Treg cells (%), median (IQR)	7.6 (6.5–8.9)	4.9 (4.1–7.0)	−6.343	<0.001
Th17/Treg ratio, median (IQR)	0.3 (0.2–0.4)	0.7 (0.5–1.1)	−9.362	<0.001

Th17, T helper 17; Treg, regulatory T; HCs, healthy controls; IQR, interquartile range. 1.

### Association of serum RBP4 with Th17/Treg balance In stroke patients and HCs

**Figure 2:**
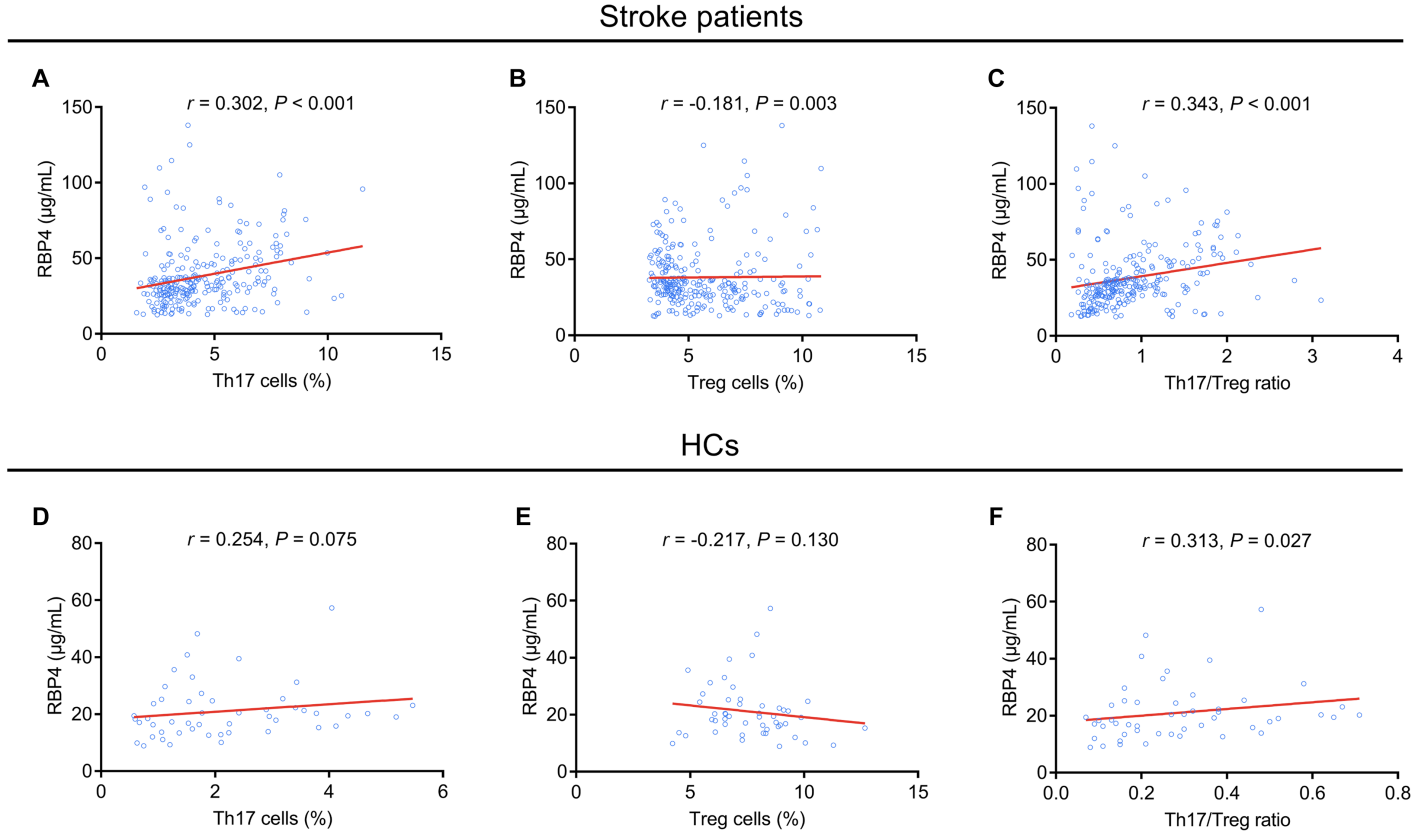
Serum RBP4 was positively associated with a Th17/Treg imbalance. Association of serum RBP4 with Th17 cells **(A)**, Treg cells **(B)**, and the Th17/Treg ratio **(C)** in stroke patients. Association of serum RBP4 with Th17 cells **(D)**, Treg cells **(E)**, and the Th17/Treg ratio **(F)** in HCs.

In stroke patients, serum RBP4 was positively associated with Th17 cells (*p* < 0.001), and the Th17/Treg ratio (*p* < 0.001) and negatively associated with Treg cells (*p* = 0.003). In addition, the correlation of serum RBP4 with Th17 cells and the Th17/Treg ratio was relatively strong, while its association with Treg cells was relatively weak (Figures 2A–C). In HCs, serum RBP4 was positively associated with the Th17/Treg ratio (*p* = 0.027), but it was not correlated with Th17 cells (*p* = 0.075) or Treg cells (*p* = 0.130) (Figures 2D–F).

### Association of serum RBP4 with characteristics of stroke patients

Increased serum RBP4 was associated with hyperlipidemia (*p* = 0.017), CVD (*p* = 0.006), and recurrent stroke (*p* = 0.011). However, it was not associated with age, gender, education level, marital status, place of residence, history of smoking, hypertension, diabetes, CKD, or lesion location (all *p* > 0.050) (Table 3).

**Table 3:** Correlation of RBP4 with characteristics of stroke patients.

Characteristics	RBP4 (μg/mL), median (IQR)	*Z* or *H*	*p* value
Age		−1.120	0.263
<60 years	29.7 (22.1–42.7)		
≥60 years	33.1 (26.2–46.4)		
Gender		−0.112	0.911
Women	32.3 (25.3–48.1)		
Men	33.2 (26.2–43.9)		
Educational level		4.178	0.124
Primary school or less	33.3 (23.6–47.7)		
Middle or high school	34.1 (27.3–48.1)		
Undergraduate or above	29.9 (25.3–35.9)		
Marital status		−0.164	0.870
Married	33.1 (26.4–42.6)		
Single/divorced/widowed	32.5 (25.4–47.5)		
Place of residence		−0.052	0.959
Urban	32.8 (25.3–45.9)		
Rural	33.3 (28.2–36.9)		
History of smoking		−1.382	0.167
No	31.2 (25.3–42.7)		
Yes	34.6 (26.9–46.1)		
Hypertension		−1.226	0.220
No	32.5 (20.3–44.7)		
Yes	33.1 (26.3–46.0)		
Hyperlipidemia		−2.381	0.017
No	31.2 (25.0–41.6)		
Yes	34.5 (26.8–51.0)		
Diabetes		−1.569	0.117
No	32.1 (25.8–42.5)		
Yes	35.0 (26.9–51.8)		
CKD		−0.806	0.420
No	32.8 (25.9–42.5)		
Yes	33.5 (26.4–49.5)		
CVD		−2.759	0.006
No	31.5 (25.3–40.8)		
Yes	35.0 (27.4–51.9)		
Lesion location		1.062	0.588
Left	32.4 (26.1–37.6)		
Right	33.3 (25.8–44.9)		
Bilateral/brainstem/unknown	33.0 (23.8–51.2)		
Diagnoses		−2.543	0.011
First stroke	31.8 (25.3–40.3)		
Recurrent stroke	36.2 (27.8–53.6)		

RBP4, Retinol binding protein 4; IQR, interquartile range; CKD, chronic kidney disease; CVD, cardiovascular disease.

The effect size of *Z* was used for the characteristics, except for educational level and lesion location, where the effect size of H was used.

### Association of serum RBP4 and Th17/Treg balance with MMSE score and cognitive impairment at enrollment in stroke patients

**Figure 3:**
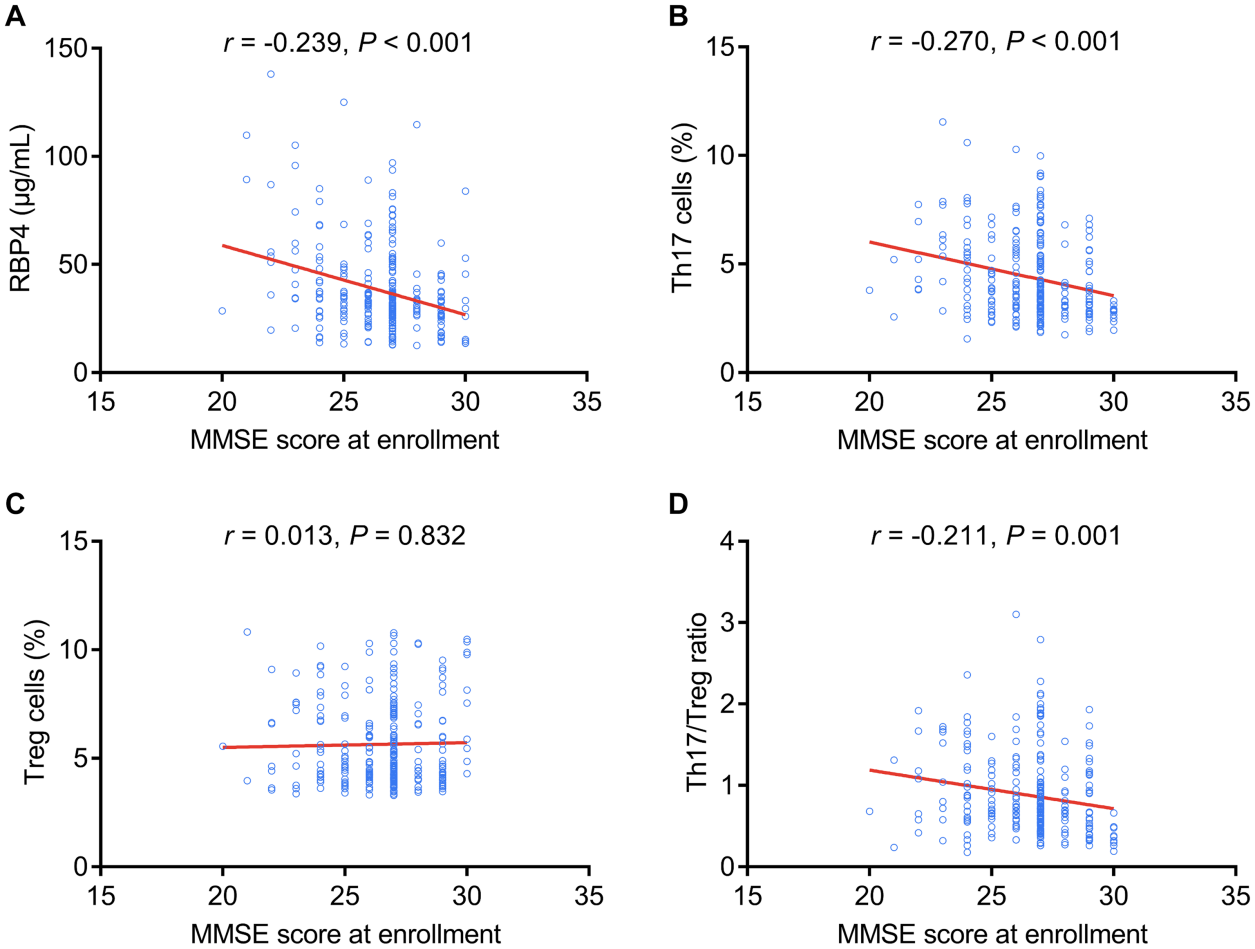
Serum RBP4 and Th17/Treg imbalances were negatively associated with the MMSE score at enrollment. Association of serum RBP4 **(A)**, Th17 cells **(B)**, Treg cells **(C)**, and the Th17/Treg ratio **(D)** with MMSE score at enrollment in stroke patients.

Serum RBP4 (*p* < 0.001), Th17 cells (*p* < 0.001), and the Th17/Treg ratio (*p* = 0.001) were negatively associated with MMSE score at enrollment, but Treg cells (*p* = 0.832) were not associated with MMSE score at enrollment in stroke patients (Figures 3A–D).

**Figure 4:**
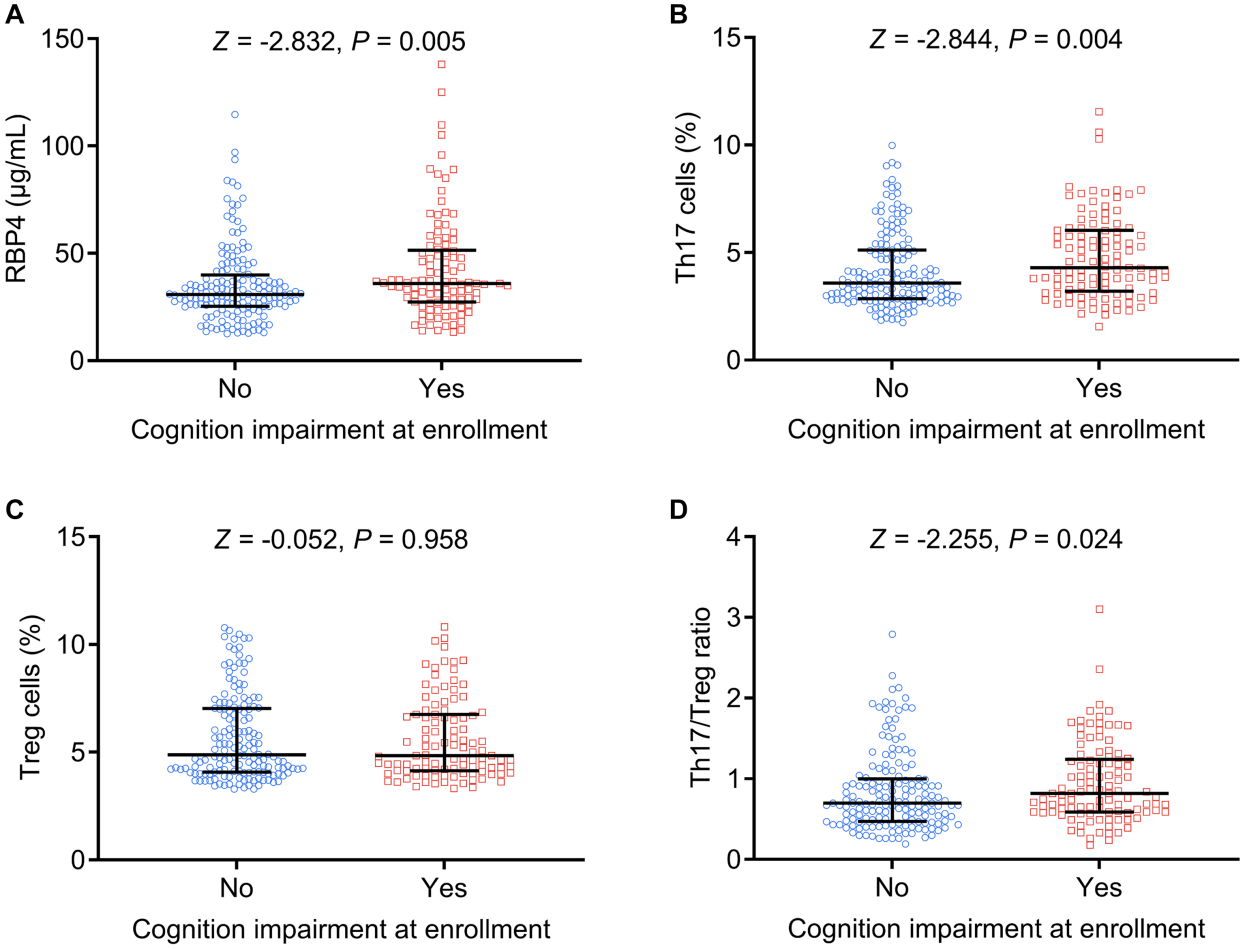
Serum RBP4 and Th17/Treg imbalances were associated with the occurrence of cognitive impairment at enrollment. Association of serum RBP4 **(A)**, Th17 cells **(B)**, Treg cells **(C)**, and the Th17/Treg ratio **(D)** with cognitive impairment at enrollment in stroke patients.

Similarly, increased serum RBP4 (*p* = 0.005), Th17 cells (*p* = 0.004), and Th17/Treg ratio (*p* = 0.024) were associated with cognitive impairment at enrollment, whereas Treg cells (*p* = 0.958) were not associated with cognitive impairment at enrollment in stroke patients (Figures 4A–D).

### Association of serum RBP4 with 1-, 2-, and 3-year MMSE scores and their decline in stroke patients

**Figure 5:**
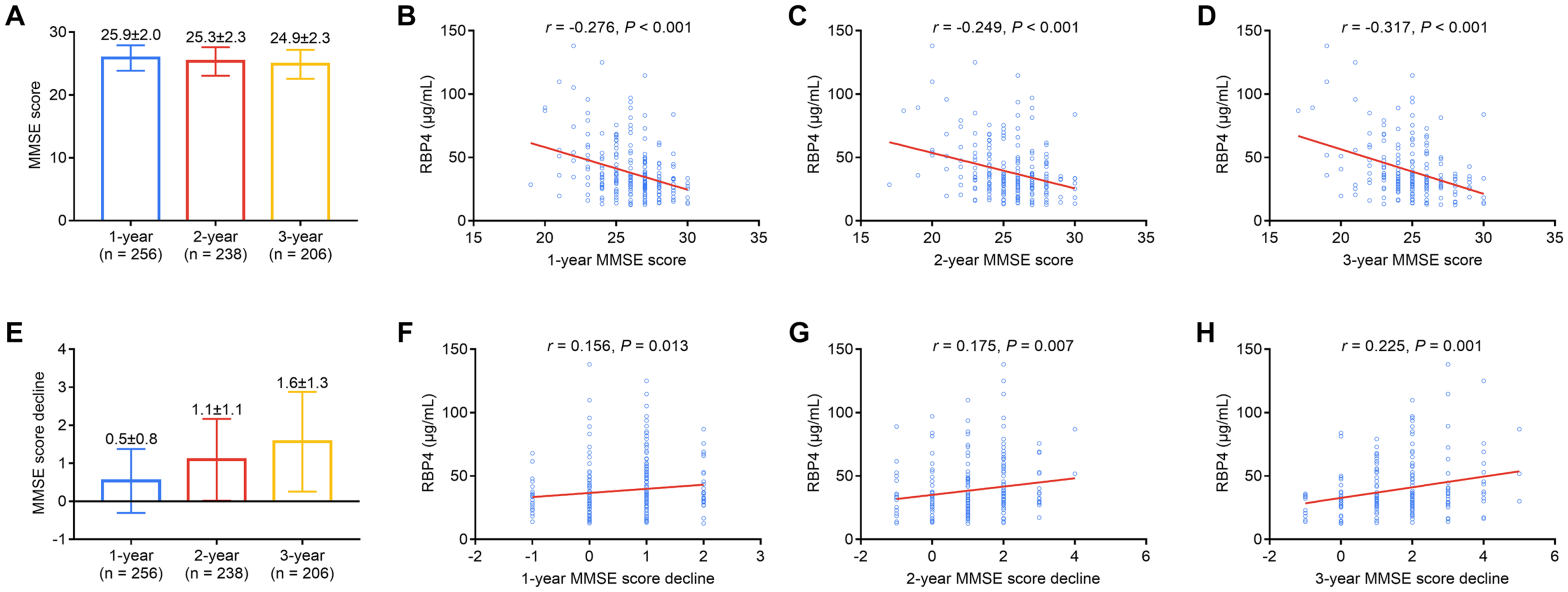
Serum RBP4 was negatively associated with 1-, 2-, and 3-year MMSE scores and positively associated with a decline in scores over 3 years. Detailed 1-, 2-, and 3-year MMSE scores in stroke patients **(A)**. Association of serum RBP4 with 1-year **(B)**, 2-year **(C)**, and 3-year **(D)** MMSE scores in stroke patients. Detailed 1-, 2-, and 3-year MMSE score decline in stroke patients **(E)**. Correlation of serum RBP4 with 1- **(F)**, 2- **(G)**, and 3-year **(H)** MMSE score decline in stroke patients.

1-, 2-, and 3-year MMSE scores in stroke patients were 25.9 ± 2.0, 25.3 ± 2.3, and 24.9 ± 2.3, respectively (Figure 5A). Serum RBP4 was negatively associated with 1- (*p* < 0.001) (Figure 5B), 2- (*p* < 0.001) (Figure 5C), and 3-year (*p* < 0.001) (Figure 5D) MMSE scores. Moreover, 1-, 2-, and 3-year MMSE score declines were 0.5 ± 0.8, 1.1 ± 1.1, and 1.6 ± 1.3, respectively (Figure 5E). Serum RBP4 was positively associated with 1- (*p* = 0.013) (Figure 5F), 2- (*p* = 0.007) (Figure 5G), and 3-year (*p* = 0.001) (Figure 5H) MMSE score decline. Further backward stepwise multivariable logistic regression models were established, which showed that higher RBP4 was independently associated with an increased risk of cognitive impairment in the 3^rd^ year (odds ratio = 1.026, *p* = 0.047) (Supplementary Table S1) and a cognitive decline over 3 years (odds ratio = 1.030, *p* = 0.013) (Supplementary Table S2) in stroke patients.

In addition, a predictive model was established using the combination of serum RBP4 and hyperlipidemia, showing some ability to estimate cognitive decline over a 3-year period (AUC = 0.671) (Supplementary Figure S2). Besides, the ROC curve showed the ability of serum RBP4 to estimate the cognitive decline in stroke patients, with an AUC value of 0.632 (Supplementary Figure S3).

## Discussion

A small body of evidence suggests that increased RBP4 expression leads to activation of the innate immune response (22, 23). For instance, one study found that RBP4 promotes macrophage-induced CD4^+^ T cell activation and Th1 polarization (22). Additionally, another study showed that RBP4 overexpression induced CD4^+^ T cell infiltration (23). Consequently, it is speculated that RBP4 may also regulate the Th17 ratio, which plays an essential role in stroke progression (24). However, the association of RBP4 with Th17 cells has rarely been reported. As a result, the present study focused on the correlation of RBP4 with Th17 cells, which revealed that serum RBP4 was positively associated with Th17 cells and the Th17/Treg ratio and negatively associated with Treg cells in stroke patients. The probable explanation is as follows: RBP4 accelerated the release of adipokines (such as leptin, adiponectin, etc.), and the latter facilitated T cell differentiation into Th17 cells, but it had less of a regulatory role in Treg differentiation (25–27). Thus, serum RBP4 was positively associated with Th17 cells and the Th17/Treg ratio but showed a weak negative association with Treg cells in stroke patients.

Apart from the positive relationship between serum RBP4 and Th17/Treg imbalance, the current study also found that increased serum RBP4 was associated with hyperlipidemia, CVD, and recurrent stroke in stroke patients. Possible explanations are: (1) RBP4 increased the level of blood lipids by inducing apolipoprotein B production (28). Therefore, elevated serum RBP4 was associated with the occurrence of hyperlipidemia in stroke patients. (2) RBP4 promoted chronic vascular inflammation by modulating several proinflammatory cytokines, such as vascular cell adhesion molecule 1 (VCAM-1), intercellular adhesion molecule 1 (ICAM-1), and interleukin-6 (29). Considering that both lipid excess and inflammation aggravate the development of atherosclerosis, serum RBP4 has been positively correlated with CVD and the diagnosis of recurrent stroke in stroke patients.

Furthermore, it is well established that the Th17/Treg imbalance exacerbates cognitive impairment (24, 30–32). For instance, one study showed that an imbalance in Th17/Treg cells exacerbates cognitive impairment by activating the STAT3 pathway (30). Another study found that Th17/Treg cells facilitate cognitive deficits in a vascular dementia rat model (31). This study showed that an elevated Th17/Treg ratio was associated with a reduced MMSE score at enrollment in stroke patients, which reflected the crucial role of the Th17/Treg imbalance in the progression of cognitive impairment.

Regarding the involvement of RBP4 in cognitive disorders, a biochemical analysis indicated that RBP4 is a potential biomarker involved in immunological and coagulation pathways in Alzheimer’s disease (33). Another study found that RBP4 concentration was positively associated with cognitive dysfunction in diabetic nephropathy patients with silent cerebral infarction (17). In contrast, a previous study showed that RBP4 is not correlated with the incidence or cognitive decline of dementia (34). This study has shown that serum RBP4 was negatively associated with baseline (at enrollment) and 1-, 2-, and 3-year MMSE scores but positively associated with 1-, 2-, and 3-year MMSE score decline in stroke patients. The possible reasons are: (1) RBP4 binding to Lp-PLA2 and netrin-1 aggravated neuronal loss and further led to cognitive impairment (17, 18). As a result, serum RBP4 was negatively associated with baseline and 1-, 2-, and 3-year MMSE scores but positively associated with 1-, 2-, and 3-year MMSE score decline in stroke patients. (2) As revealed by the correlation analysis between serum RBP4 and Th17/Treg imbalance in this study, RBP4 may exacerbate cognitive impairment by positively modulating Th17/Treg imbalance, while further verification of this hypothesis is warranted.

This study first demonstrated the positive correlation between serum RBP4 and Th17 cells and 3-year cognitive impairment in stroke patients. However, the present study had several limitations: (1) This was a single-center study, and selective bias existed. Further multi-center is necessary to validate the findings. (2) PB samples were collected only at patient enrollment, and the variation of serum RBP4 in stroke patients remains unclear. (3) Although this study revealed a positive correlation between serum RBP4 and Th17/Treg imbalance in stroke patients, its underlying mechanism requires further exploration. (4) The 3-year follow-up of the present study was relatively short to estimate the predictive value of RBP4 for cognitive decline, and long-term studies are needed to further validate the findings. (5) Th2 cells participated in the cognitive impairment of stroke patients according to the previous study (35), but the correlation between serum RBP4 and Th2 cells was not explored in the current study.

## Conclusion

In summary, elevated serum RBP4 is associated with Th17/Treg imbalance and aggravated 1-, 2-, and 3-year MMSE score decline, with the potential to reflect cognitive impairment in stroke patients to some extent. Consequently, early and timely interventions and physical rehabilitation are more necessary in stroke patients with high serum RBP4.

## Data availability statement

The original contributions presented in the study are included in the article/Supplementary material, further inquiries can be directed to the corresponding author.

## Ethics statement

This study was approved by the Ethics Committee of Fuyang People’s Hospital, and informed consent was obtained from each participant or guardian.

## Author contributions

FW and YQ performed the data collection and statistical analysis, wrote the article, and critically revised it. ZL performed the conception and design, wrote the article, and critically revised the article. All authors contributed to the article and approved the submitted version.

## Conflict of interest

The authors declare that the research was conducted in the absence of any commercial or financial relationships that could be construed as a potential conflict of interest.

## Publisher’s note

All claims expressed in this article are solely those of the authors and do not necessarily represent those of their affiliated organizations, or those of the publisher, the editors and the reviewers. Any product that may be evaluated in this article, or claim that may be made by its manufacturer, is not guaranteed or endorsed by the publisher.

## References

[1] Hankey GJ. Stroke. Lancet. (2017) 389:641–54. doi: 10.1016/S0140-6736(16)30962-X 27637676

[2] Thayabaranathan T Kim J Cadilhac DA Thrift AG Donnan GA Howard G. Global stroke statistics 2022. Int J Stroke. (2022) 17:946–56. doi: 10.1177/17474930221123175, PMID: 35975986 PMC9980380

[3] Wu S Wu B Liu M Chen Z Wang W Anderson CS. Stroke in China: advances and challenges in epidemiology, prevention, and management. Lancet Neurol. (2019) 18:394–405. doi: 10.1016/S1474-4422(18)30500-3, PMID: 30878104

[4] Phipps MS Cronin CA. Management of Acute Ischemic Stroke. BMJ. (2020) 368:l6983. doi: 10.1136/bmj.l6983 32054610

[5] Kuriakose D Xiao Z. Pathophysiology and treatment of stroke: present status and future perspectives. Int J Mol Sci. (2020) 21:21207609. doi: 10.3390/ijms21207609 PMC7589849 33076218

[6] Gil-Garcia CA Flores-Alvarez E Cebrian-Garcia R Mendoza-Lopez AC Gonzalez-Hermosillo LM Garcia-Blanco MD. Essential topics about the imaging diagnosis and treatment of hemorrhagic stroke: a comprehensive review of the 2022 aha guidelines. Curr Probl Cardiol. (2022) 47:101328. doi: 10.1016/j.cpcardiol.2022.101328, PMID: 35870549

[7] Rost NS Brodtmann A Pase MP van Veluw SJ Biffi A Duering M. Post-stroke cognitive impairment and dementia. Circ Res. (2022) 130:1252–71. doi: 10.1161/CIRCRESAHA.122.319951 35420911

[8] Huang YY Chen SD Leng XY Kuo K Wang ZT Cui M. Post-stroke cognitive impairment: epidemiology, risk factors, and management. J Alzheimers Dis. (2022) 86:983–99. doi: 10.3233/JAD-215644, PMID: 35147548

[9] Katan M Luft A. Global burden of stroke. Semin Neurol. (2018) 38:208–11. doi: 10.1055/s-0038-1649503 29791947

[10] Quinn TJ Richard E Teuschl Y Gattringer T Hafdi M O'Brien JT. European stroke organisation and European academy of neurology joint guidelines on post-stroke cognitive impairment. Eur J Neurol. (2021) 28:3883–920. doi: 10.1111/ene.15068, PMID: 34476868

[11] Zhou W Ye SD Wang W. Elevated retinol binding protein 4 levels are associated with atherosclerosis in diabetic rats via Jak2/Stat3 signaling pathway. World J Diabetes. (2021) 12:466–79. doi: 10.4239/wjd.v12.i4.466 33889291 PMC8040077

[12] Ji Y Song J Su T Gu X. Adipokine retinol binding protein 4 and cardiovascular diseases. Front Physiol. (2022) 13:856298. doi: 10.3389/fphys.2022.856298 35309061 PMC8924404

[13] Olsen T Blomhoff R. Retinol, retinoic acid, and retinol-binding protein 4 are differentially associated with cardiovascular disease, type 2 diabetes, and obesity: an overview of human studies. Adv Nutr. (2020) 11:644–66. doi: 10.1093/advances/nmz131 31868199 PMC7231588

[14] Liu Y Zhong Y Chen H Wang D Wang M Ou JS. Retinol-binding protein-dependent cholesterol uptake regulates macrophage foam cell formation and promotes atherosclerosis. Circulation. (2017) 135:1339–54. doi: 10.1161/CIRCULATIONAHA.116.024503, PMID: 28122883

[15] Zhu YY Zhang JL Liu L Han Y Ge X Zhao S. Evaluation of serum retinol-binding Protein-4 levels as a biomarker of poor short-term prognosis in ischemic stroke. Biosci Rep. (2018) 38:BSR20180786. doi: 10.1042/BSR20180786 30038059 PMC6131228

[16] Liu S Li J Rong X Wei Y Peng Y Shen Q. Plasma retinol binding protein 4 as a biomarker for detecting progressive stroke and prediction of early prognosis in patients with acute ischemic stroke. Curr Neurovasc Res. (2021) 18:381–8. doi: 10.2174/1567202618666211122150730 34809546

[17] Chen D Huang X Lu S Deng H Gan H du X. Rbp4/Lp-Pla2/Netrin-1 signaling regulation of cognitive dysfunction in diabetic nephropathy complicated with silent cerebral infarction. Exp Clin Endocrinol Diabetes. (2017) 125:547–53. doi: 10.1055/s-0043-109099, PMID: 28704853

[18] Buxbaum JN Roberts AJ Adame A Masliah E. Silencing of murine transthyretin and retinol binding protein genes has distinct and shared behavioral and neuropathologic effects. Neuroscience. (2014) 275:352–64. doi: 10.1016/j.neuroscience.2014.06.019 24956283

[19] Goodman AB. Retinoid receptors, transporters, and metabolizers as therapeutic targets in late onset Alzheimer disease. J Cell Physiol. (2006) 209:598–603. doi: 10.1002/jcp.20784 17001693

[20] Powers WJ Rabinstein AA Ackerson T Adeoye OM Bambakidis NC Becker K. Guidelines for the early Management of Patients with acute ischemic stroke: 2019 update to the 2018 guidelines for the early Management of Acute Ischemic Stroke: a guideline for healthcare professionals from the American Heart Association/American Stroke Association. Stroke. (2019) 50:e344–418. doi: 10.1161/STR.0000000000000211, PMID: 31662037

[21] Wang C Huo H Li J Zhang W Liu C Jin B. The longitudinal changes of serum Jkap and il-17a, and their linkage with anxiety, depression, and cognitive impairment in acute ischemic stroke patients. J Clin Lab Anal. (2022) 36:e24762. doi: 10.1002/jcla.24762, PMID: 36397283 PMC9756983

[22] Moraes-Vieira PM Castoldi A Aryal P Wellenstein K Peroni OD Kahn BB. Antigen presentation and T-cell activation are critical for Rbp4-induced insulin resistance. Diabetes. (2016) 65:1317–27. doi: 10.2337/db15-1696 26936962 PMC4839203

[23] Moraes-Vieira PM Yore MM Dwyer PM Syed I Aryal P Kahn BB. Rbp4 activates antigen-presenting cells, leading to adipose tissue inflammation and systemic insulin resistance. Cell Metab. (2014) 19:512–26. doi: 10.1016/j.cmet.2014.01.018 24606904 PMC4078000

[24] Lu T Ma L Xu Q Wang X. Blood Th17 cells and il-17a as candidate biomarkers estimating the progression of cognitive impairment in stroke patients. J Clin Lab Anal. (2022) 36:e24581. doi: 10.1002/jcla.24581 35808926 PMC9396181

[25] Sun X Feng X Tan W Lin N Hua M Wei Y. Adiponectin exacerbates collagen-induced arthritis via enhancing Th17 response and prompting Rankl expression. Sci Rep. (2015) 5:11296. doi: 10.1038/srep11296, PMID: 26063682 PMC4462752

[26] Vollmer CM Dias ASO Lopes LM Kasahara TM Delphim L Silva JCC. Leptin favors Th17/Treg cell subsets imbalance associated with allergic asthma severity. Clin Transl Allergy. (2022) 12:e12153. doi: 10.1002/clt2.12153, PMID: 35734271 PMC9194742

[27] Swiderska M Jaroszewicz J Stawicka A Parfieniuk-Kowerda A Chabowski A Flisiak R. The interplay between Th17 and T-regulatory responses as well as adipokines in the progression of non-alcoholic fatty liver disease. Clin Exp Hepatol. (2017) 3:127–34. doi: 10.5114/ceh.2017.68466 29062902 PMC5649483

[28] Liu Y Chen H Wang J Zhou W Sun R Xia M. Elevated retinol binding protein 4 induces apolipoprotein B production and associates with hypertriglyceridemia. J Clin Endocrinol Metab. (2015) 100:E720–8. doi: 10.1210/jc.2015-1072 25781360

[29] Zabetian-Targhi F Mahmoudi MJ Rezaei N Mahmoudi M. Retinol binding protein 4 in relation to diet, inflammation, immunity, and cardiovascular diseases. Adv Nutr. (2015) 6:748–62. doi: 10.3945/an.115.008292 26567199 PMC4642414

[30] Zhang X Zhang X Qiu C Shen H Zhang H He Z. The imbalance of Th17/Treg via Stat3 activation modulates cognitive impairment in *P. gingivalis* Lps-induced periodontitis mice. J Leukoc Biol. (2021) 110:511–24. doi: 10.1002/JLB.3MA0521-742RRR, PMID: 34342041

[31] Qiuping L Pan P Zhenzhen L Zhen Z Xuezhu Z Shuting L. Acupuncture regulates the Th17/Treg balance and improves cognitive deficits in a rat model of vascular dementia. Heliyon. (2023) 9:e13346. doi: 10.1016/j.heliyon.2023.e13346 36816326 PMC9929319

[32] Zhang Y Liu M Sun H Yin K. Matrine improves cognitive impairment and modulates the balance of Th17/Treg cytokines in a rat model of Abeta1-42-induced Alzheimer's disease. Cent Eur J Immunol. (2015) 40:411–9. doi: 10.5114/ceji.2015.56961 26862304 PMC4737738

[33] Nielsen JE Honoré B Vestergård K Maltesen RG Christiansen G Bøge AU. Shotgun-based proteomics of extracellular vesicles in Alzheimer's disease reveals biomarkers involved in immunological and coagulation pathways. Sci Rep. (2021) 11:18518. doi: 10.1038/s41598-021-97969-y, PMID: 34531462 PMC8445922

[34] Ishii M Kamel H Iadecola C. Retinol binding protein 4 levels are not altered in preclinical Alzheimer's disease and not associated with cognitive decline or incident dementia. J Alzheimers Dis. (2019) 67:257–63. doi: 10.3233/JAD-180682 30562901 PMC6385158

[35] Yu S Cui W Han J Chen J Tao W. Longitudinal change of Th1, Th2, and Th17 cells and their relationship between cognitive impairment, stroke recurrence, and mortality among acute ischemic stroke patients. J Clin Lab Anal. (2022) 36:e24542. doi: 10.1002/jcla.24542 35689536 PMC9280005

