# Supplementary material for: Serum retinol-binding protein 4 in stroke patients: correlation with T helper 17/regulatory T cell imbalance and 3-year cognitive function decline
Source: Front Neurol. 2023 Sep 21;14:1217979. doi: 10.3389/fneur.2023.1217979 (PMC10551125; doi:10.3389/fneur.2023.1217979)
Supplement: Supplementary file 1 [file Table_1.docx]

**Supplementary Table 1.** Backward stepwise multi-variable logistics regression model on 3-year cognition impairment of stroke patients.

| Factors | OR (95% CI) | *P* value |
| --- | --- | --- |
| Higher RBP4 | 1.026 (1.001-1.053) | 0.047 |
| Education level |  |  |
| Primary school or below (reference) | 1.000 | (-) |
| Middle or high school | 1.018 (0.426-2.432) | 0.968 |
| Undergraduate or above | 0.333 (0.122-0.909) | 0.032 |
| Hyperlipidemia | 2.549 (1.142-5.690) | 0.022 |
| Recurrent stroke | 2.502 (0.947-6.610) | 0.064 |

OR, odds ratio; CI, confidence interval; RBP4, Retinol binding protein 4.
