# Supplementary material for: Serum retinol-binding protein 4 in stroke patients: correlation with T helper 17/regulatory T cell imbalance and 3-year cognitive function decline
Source: Front Neurol. 2023 Sep 21;14:1217979. doi: 10.3389/fneur.2023.1217979 (PMC10551125; doi:10.3389/fneur.2023.1217979)
Supplement: Supplementary file 2 [file Table_2.docx]

**Supplementary Table 2.** Backward stepwise multi-variable logistics regression model on 3-year cognitive decline of stroke patients.

| Factors | OR (95% CI) | *P* value |
| --- | --- | --- |
| Higher RBP4 | 1.030 (1.006-1.054) | 0.013 |
| Hyperlipidemia | 1.886 (0.928-3.835) | 0.080 |

OR, odds ratio; CI, confidence interval; RBP4, Retinol binding protein 4.
